# Methylation levels assessment with Methylation-Sensitive High-Resolution Melting (MS-HRM)

**DOI:** 10.1371/journal.pone.0273058

**Published:** 2022-09-06

**Authors:** Sally Samsø Mathiasen, Jan Bińkowski, Tina Kjeldsen, Tomasz K. Wojdacz, Lise Lotte Hansen

**Affiliations:** 1 Department of Biomedicine, Aarhus University, Aarhus, Denmark; 2 Independent Clinical Epigenetics Laboratory, Pomeranian Medical University in Szczecin, Szczecin, Poland; Dartmouth College Geisel School of Medicine, UNITED STATES

## Abstract

Testing for disease-related DNA methylation changes provides clinically relevant information in personalized patient care. Methylation-Sensitive High-Resolution Melting (MS-HRM) is a method used for measuring methylation changes and has already been used in diagnostic settings. This method utilizes one set of primers that initiate the amplification of both methylated and non-methylated templates. Therefore, the quantification of the methylation levels using MS-HRM is hampered by the PCR bias phenomenon. Some approaches have been proposed to calculate the methylation level of samples using the high-resolution melting (HRM) curves. However, limitations of the methylation calculation using MS-HRM have not been evaluated systematically and comprehensively. We used the Area Under the Curve (AUC), a derivative of the HRM curves, and least square approximation (LSA) to establish a procedure that allowed us to infer methylation levels in an MS-HRM experiment and assess the limitations of that procedure for the assays’ specific methylation level measurement. The developed procedure allowed, with certain limitations, estimation of the methylation levels using HRM curves.

## Introduction

Enzymatic addition of methyl groups to cytosines in the DNA molecules is referred to as DNA-methylation. In humans, this modification almost exclusively occurs within CpG dinucleotides. DNA-methylation is one of the major mechanisms of gene expression regulation and in general terms genes with methylated promoters are not expressed. Methylation changes in gene promoters have long been shown to play a key role, if not initiate, neoplastic transformation and the evidence for critical significance of methylation changes in etiology of other diseases is increasing exponentially. At the same time, it has already become a paradigm that the disease related methylation changes are useful biomarkers at all stages of clinical disease management from risk assessment through early diagnosis and treatment personalization to post treatment management of chronic diseases [1]. Nevertheless, despite increasing evidence for the clinical significance of the methylation changes, there is still no consensus in the field regarding the technology most suitable for the diagnostic testing of methylation biomarkers.

Methylation-Sensitive High-Resolution Melting (MS-HRM) is a PCR based method that utilizes methylation independent primers (MIP-primers) for amplification of a bisulfite modified template. In the MS-HRM protocol, the methylation changes are determined in a post-PCR manner using high-resolution melting [2]. Overall, technologies that utilize one primer set for the amplification of both methylated and non-methylated templates (MIP-primers) are hampered by the PCR bias phenomenon that significantly influences the sensitivity and accuracy of the methylation detection [3, 4]. In our previous work, we developed a primer design system that allowed us to reverse PCR bias and significantly increase the sensitivity of the detection of methylation [5]. However, this approach limits the dynamic range of the methylation levels quantification, especially in the applications where high sensitivity of methylation detection is critical.

Here, we present a comprehensive assessment of the limitations of the methylation levels estimation using MS-HRM. Additionally, we present a procedure that, with certain limitations, allowed us to calculate the methylation levels from high-resolution melting curves.

## Materials and methods

The protocol described in this peer-reviewed article is published on protocols.io, https://dx.doi.org/10.17504/protocols.io.n2bvj6yjxlk5/v1 and is included for printing as S1 File with this article.

### MS-HRM data

To generate HRM data for the development of our data analysis approach, we used four EpiMelt assays, targeting the *APC*, *BRCA1*, *H19*, and *MGMT* genes (provided by MethylDetect ApS, Aalborg, Denmark). A reference range of controls with different methylation levels was generated. The provided assays included a methylated and non-methylated control. They were mixed to gain a dilution range of 0, 0.1, 1, 5, 10, 20, 30, 40, 50, 60, 70, 80, 90, 100% of the methylated control in a non-methylated background. PCR amplification was performed using LightCycler® 480 High Resolution Melting Master (catalogue number: 04909631001) and Roche LightCycler480 platform (Roche Applied Science, Laval, PQ, Canada). Briefly, the PCR amplification mix contained: 3 mM MgCl_2,_ 1 x LightCycler® High Resolution Melting Master (Roche), 500 nM of the primer mix, and 6 μL of the provided control mix in a total volume of 20 μL. All the reactions were run in triplicates.

### Data processing

**Fig 1:**
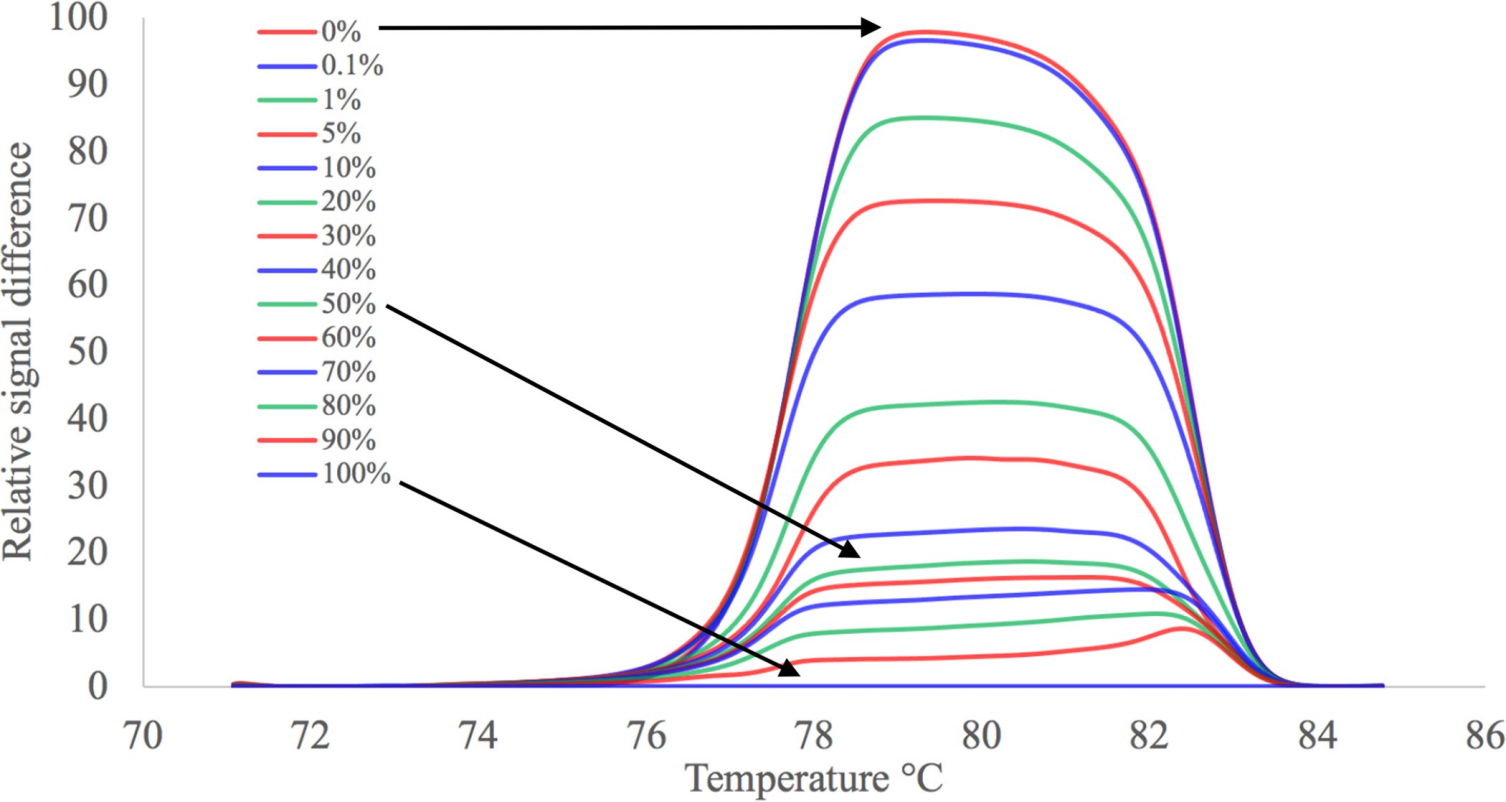
An example of the HRM melting curves transformed into a difference plot with the 100% methylation control used as a baseline for the EpiMelt *MGMT* assay. Each curve represents melting of the PCR product obtained from the specific mix of the controls (as indicated in the panel) in the range from 0.1 to 90% methylated template in non-methylated background.

The HRM curves were normalized using Gene Scanning software (an integrated part of the Roche LightCycler480 software) with default settings for each run. The difference plots were generated for each normalized melting curve with the 100% methylation controls melting curve as the baseline (Fig 1).

The difference plots were exported as a text file from the Roche LightCycler480 platform and imported to Excel (Microsoft Office, 2021). In Excel, the Area Under the Curve (AUC), and the difference plots, was calculated by summarizing the relative signal difference from each fluorescence measurement point over the temperature gradient. AUC, obtained for each control mixes with known methylation levels, was then plotted to generate standard curves. The standard curves describe the association between AUC and the methylation levels for the specific MS-HRM assay at given experimental conditions. The best association between AUC and the methylation level was tested using three equations. Eq 1) A linear association, Eq 2) exponential association with one constant (M), and Eq 3) exponential association with two constants (M and N). Least Square Approximation (LSA) was used to determine the line of best fit between the data obtained in each of the specific experiments and the proposed equation. This line then best describes the association between AUC and methylation levels and is referred to as the standard curve. Subsequently, the validation of the standard curve was tested using linear correlation analysis between obtained AUC values and calculated AUC values.

## Results and discussion

We have automated the described below data analysis procedure and this analysis can be performed using S2 File and step by step data analysing protocol described in: S1 File.

### Methylation levels and AUC do not display monotonic association in MS-HRM experiment

AUC reflects the methylation level in given samples. Following the standard approach in quantitative research, we used standard curve calculation to infer methylation levels from AUC obtained for each control mixes in the given experiment. As in previous reports [6], we initially assumed that the methylated and non-methylated template are amplified with the same efficiency, regardless of the proportion of these two templates in the sample. Therefore, we described the correlation between AUC and the methylation level as:

$$AUC(x)=AUC_{\left(0\%\right)}\cdot (1‐x)$$ (1)

**Fig 2:**
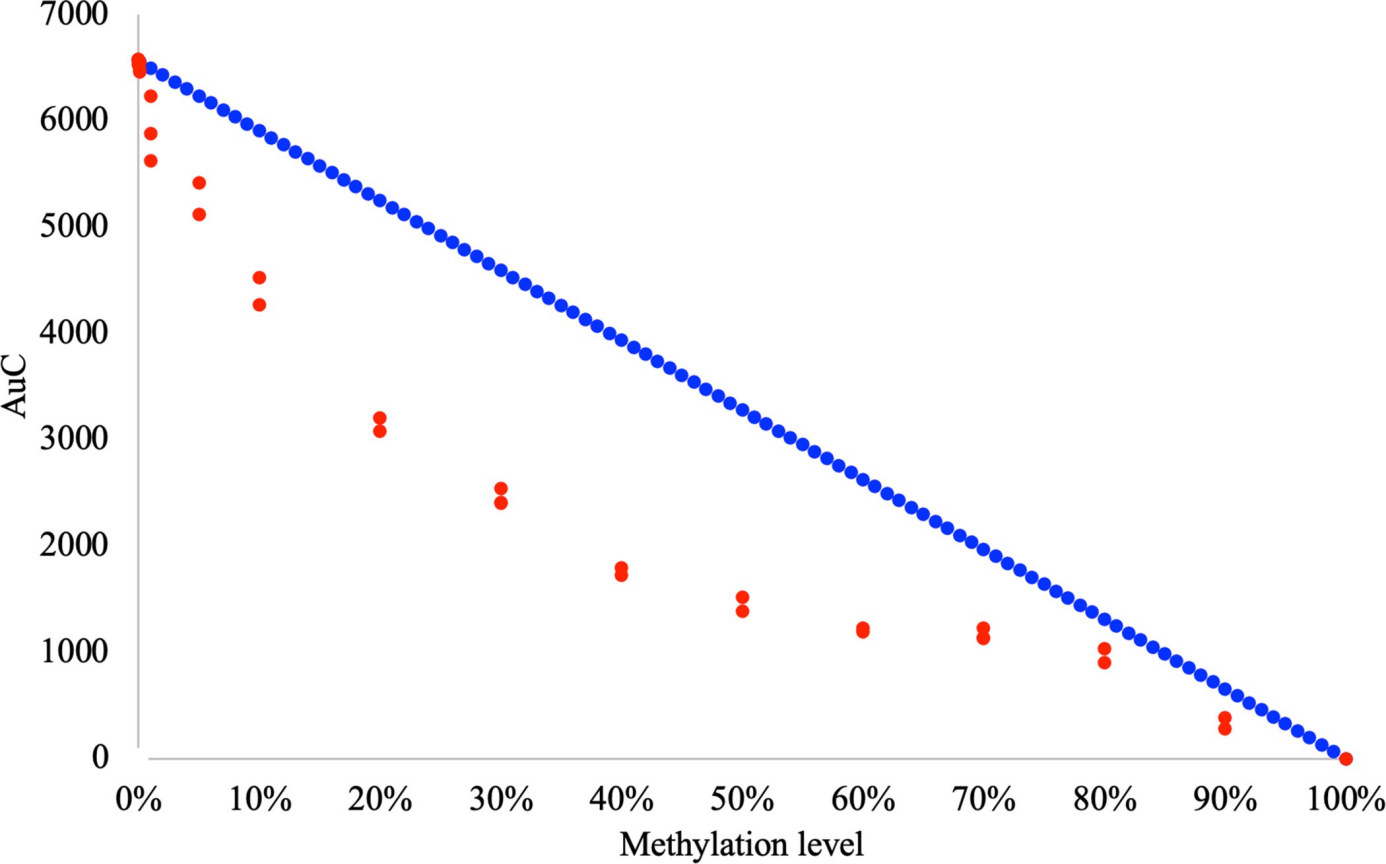
Comparison of the standard curve calculated using Eq 1 and observed AUC for each of the control mixes in the experiment for the EpiMelt *MGMT* assay. The AUC, reflecting methylation levels for each of the control mixes (red dots–triplicates for each of the control mixes) does not overlap with the standard curve (blue) calculated using Eq 1. Eq 1 describes unbiased amplification of the methylation from each of the control mixes. Overall comparison of the standard curve and AUC values obtained for each of the control mixes, indicates a significant effect of the experimental conditions and PCR bias on the amplification of the methylated and non-methylated template in each of the control mixes.

Where AUC at a given methylation level (x) was calculated by multiplying the maximum AUC_(0%)_ (AUC of the HRM curve obtained for the 0% methylation control) by the amount of non-methylated DNA (1-x) (as previously described in [6]). In Fig 2, the blue curve depicts the standard curve obtained for the EpiMelt *MGMT* assay with the above assumption. We then plotted the values of the AUC obtained for each of the control mixes in our experiment (Fig 2 - red dots (in triplicates) for each control mix). As shown in Fig 2, the results indicated that the association between AUC and methylation level was not linear as only controls with a methylation level above 80% had an observed AUC values that overlapped with the predicted standard curve.

The poor association between the observed AUC, calculated by summarizing the relative signal difference from each fluorescence measurement point over the temperature gradient (Fig 2 red), and AUC, calculated by the standard curve generated by Eq 1 (Fig 2 blue), is most likely attributed to the PCR bias phenomenon. Moreover, as the discrepancies between the standard curve and the observed AUC are not constant for all the control mixes, the PCR bias appears to depend on the proportion of methylated to non-methylated template in the PCR.

### Correction of methylation level estimate for the effect of PCR bias

Detailed analysis of the data, shown in Fig 2, indicates that the association between AUC and methylation level was non-monotonic and non-linear. Therefore, a standard curve calculated with the assumption of linear association between AUC and methylation level (blue in Fig 2) overestimates the methylation level in the control mixes from above 0% to around 80%. The observed AUC is lower than the calculated AUC for the control mixes (red dots in Fig 2). The results in Fig 2 also indicate that the underestimation of the methylation appears to be exponential. To generate a standard curve, which takes this type of association into account, we included an exponential component in Eq 1. Specifically, AUC at a given methylation level (x) was calculated by multiplying the maximum AUC_(0%)_ by the amount of non-methylated DNA (1-x) and a decreasing exponential component e^(-M·x)^ (M is a constant value describing the effect of PCR bias on amplification of the methylated template (x) in a specific experiment).


$$AUC(x)=AUC_{\left(0\%\right)}\cdot (1‐x)\cdot e^{\left(‐M\cdot x\right)}$$ (2)

**Fig 3:**
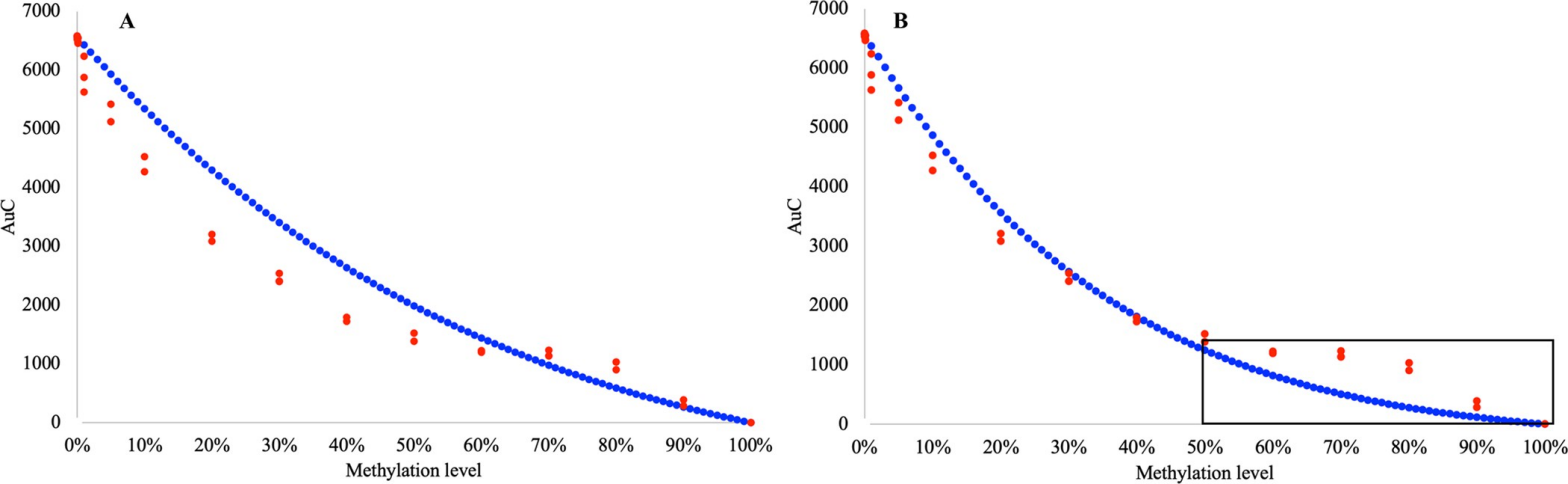
Comparison of the standard curve calculated using Eq 2 and observed AUC for each of the control mixes in the experiment for the EpiMelt *MGMT* assay. (A) The standard curve (blue) describes the association relationship between AUC and methylation level calculated using Eq 2 and observed values of the AUC (red dots–triplicates for each of the control mixes) with M-value set to 1 for the EpiMelt *MGMT* assay. (B) The standard curve with M-value was calculated for this specific experiment with LSA. The black box shows the region with the highest discrepant regarding estimation of AUC values.

Fig 3A shows the standard curve generated using Eq 2 when the M value is 1. The association between the observed AUC, calculated by summarizing the relative signal difference from each fluorescence measurement point over the temperature gradient (Fig 3A red), and AUC, calculated by the standard curve generated by Eq 2 (Fig 3A blue), is better compared to the association using Eq 1. Thus, the standard curve generated using Eq 2 describes the observed association between AUC and methylation levels in control mixes significantly more accurate. We then used Least Squares Approximation (LSA) to find the value for M (in this specific experiment) for which Eq 2 would best fit the empirical data obtained from the control mixes. The standard curve generated using LSA and M value calculated for this experimental data is described in Fig 3B. This curve significantly better reflects the association between AUC and methylation level in this experiment. It is also clear from Fig 3B that at high proportions of the methylated template in the sample, the discrepancies between the generated standard curve and the observed AUC in the control mixes, suggest over-amplification of the non-methylated template (Fig 3B grey box), the observed AUC is larger than the calculated AUC for the control mixes.

The closer analysis of discrepancies between data from expected and calculated methylation levels at high concentrations of the methylated template suggested that, at this methylation level, the non-methylated template was overestimated (black box Fig 3B). Therefore, we implemented constant N in the equation to model the influence of the PCR bias on amplification of the non-methylated template (1-x)

$$AUC(x)=AUC_{\left(0\%\right)}\cdot (1‐x)\cdot e^{\left(‐M\cdot x)\cdot (N\cdot (1‐x)\right)}$$ (3)

**Fig 4:**
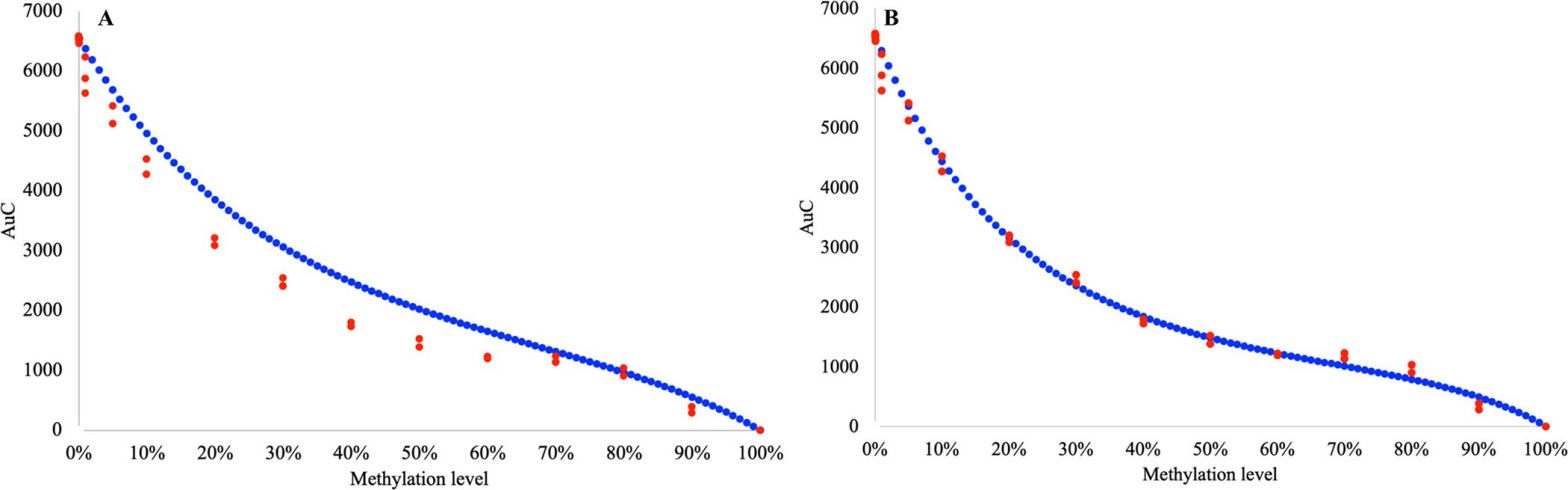
Comparison of the standard curve calculated using Eq 3 and observed AUC for each of the control mixes in the experiment for the EpiMelt *MGMT* assay. The standard curve (blue) describing the association relationship between AUC and methylation level calculated using Eq 3, and the observed values of AUC (red dots–triplicates for each of the control mixes) for the EpiMelt *MGMT* assay. (A) with M and N values set to 1 and (B) the standard curve with M-and N values calculated for this specific experiment with LSA.

Again, the standard curve with N, set to 1 (N = 1), better described the empirical methylation level (Fig 4B) and the use of LSA to calculate the precise value of N for this experiment, significantly improved the accuracy of the standard curve (Fig 4C).

**Fig 5:**
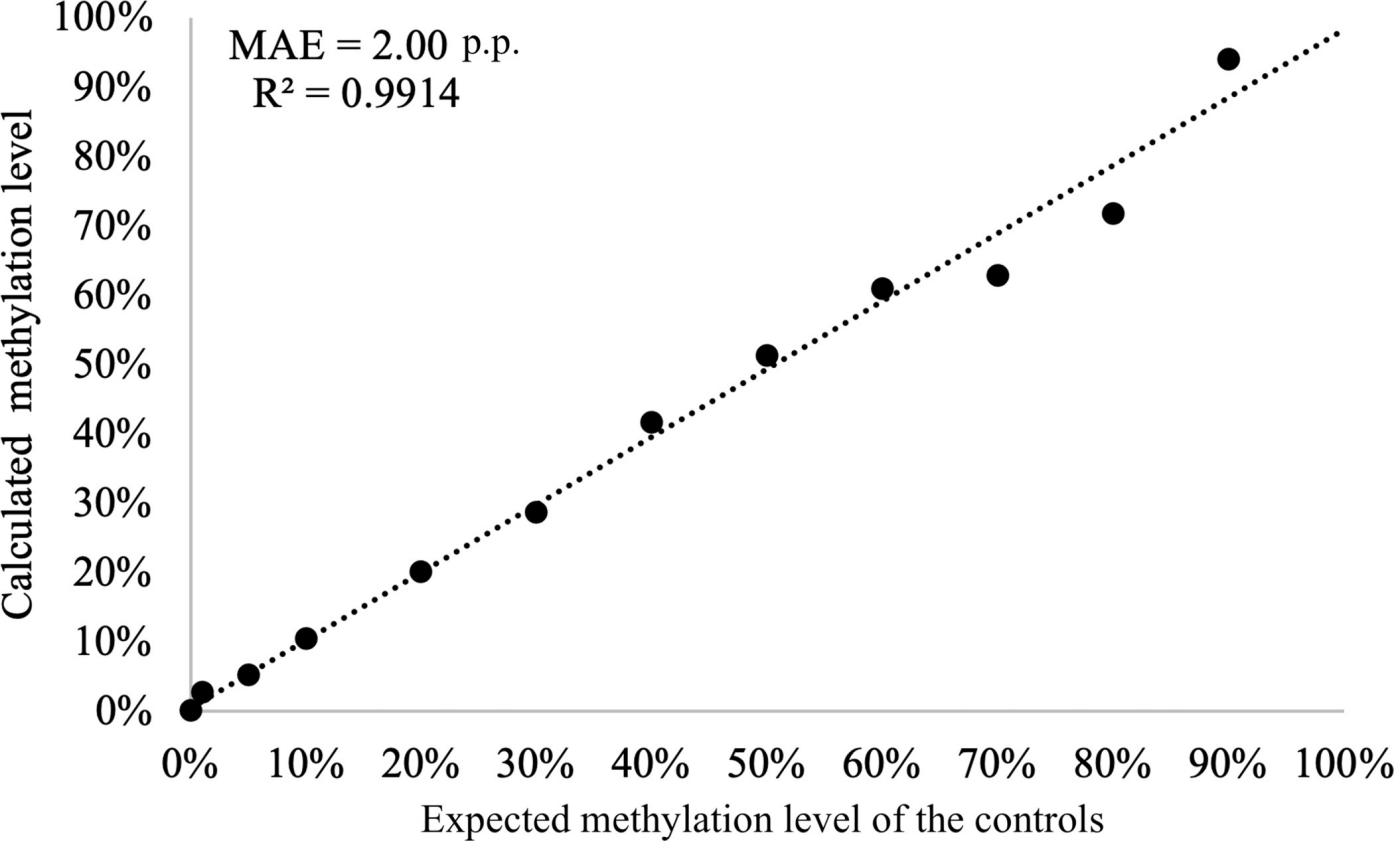
Goodness of fit analyses using the Eq 3. The AUC predicted by the Eq 3 and empirical data in this experiment fit with R^2^ = 0.9914 and MAE = 2.00 percent point. All data points were plotted as a mean of triplicate samples.

Finally, we used linear regression to test the association between the observed and expected methylation levels obtained using Eq 3 using the LSA procedure. The R^2^ = 0.9914 of this comparison was and the mean absolute error (MAE) = 2.00 percent point (p.p.), indicating a good fit between the tested data sets. (Fig 5).

We performed identical analyses for three additional EpiMelt assays, targeting the *APC*, *BRCA1* and *H19* genes. The following analyses showed similar results to the ones presented here. For details (See S4–S7 Files).

### Validation of the new quantification procedure

**Fig 6:**
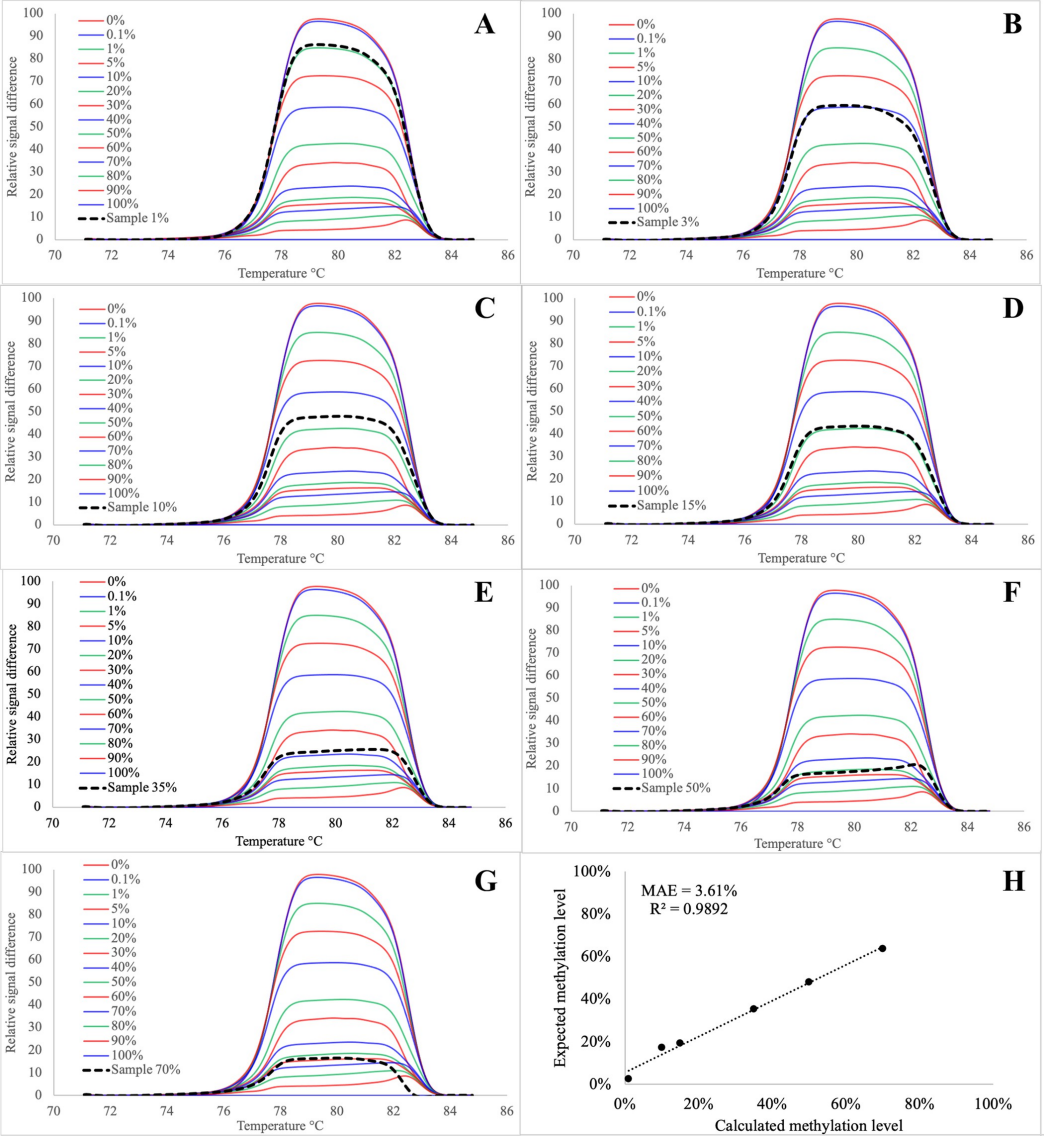
Example of the difference plots obtained for control mixes and constructed mixes for the EpiMelt *MGMT* assays with 100% methylation control as reference (panels A to F) and goodness of fit analysis for this assay (panel H). In each panel the red, blue, and green curves represent difference plots for the control mixes and black curve is a difference plot for the constructed mix of methylated template in non-methylated background (in Panel A = 1%, B = 3%, C = 10%, D = 15%, E = 35%, F = 50% and G = 70%). (H) Goodness of fit test for this assay resulted in: R^2^ = 0.9892 and MAE = 3.61%. All data points were plotted as a mean of triplicate samples.

We then evaluated the accuracy of the new quantification approach by constructing seven independent mixes of methylated templates in a non-methylated background with methylation levels of 1, 3, 10, 15, 35, 50, and 70%, for the EpiMelt *MGMT* assay, and calculated the methylation levels for those samples using the described procedure. Fig 6A–6G show the difference plots for each normalized melting curve with the 100% methylation curve as the baseline as well as one of the seven constructed mixes: pink curves in panel A-G show the constructed mix with panel A = 1%, panel B = 3%, panel C = 10%, panel D = 15%, panel E = 30%, panel F = 50% and panel G = 70% methylation level.

In general, the difference plot curves, observed for the constructed mixes, overlapped with the curves obtained from the control mixes representing the most similar proportion of methylated to non-methylated template used in each of the experiments (Fig 6), apart from the 3% methylation level mix, which overlapped the control mix of 10% (Fig 6B). We then calculated the methylation levels in those samples using Eq 3. The results are provided in Table 1, where the difference between the calculated (using Eq 3) and expected methylation level for each of the independent mixes is shown in the far-right column. In general, we observed an MAE of 3.61 p.p. between the calculated and expected methylation levels, which is a rather acceptable discrepancy margin in this type of studies.

**Table 1:** The methylation levels calculated for each of the independent mixes used in the experiment.

Sample name	Expected methylation level	Calculated methylation level	Variation between expected and calculated methylation levels (residuals)
Sample 70%	70%	64%	6 p.p.
Sample 50%	50%	48%	2 p.p.
Sample 30%	35%	35%	0 p.p.
Sample 15%	15%	19%	4 p.p.
Sample 10%	10%	17%	7 p.p.
Sample 1%	1%	3%	2 p.p.

Mean absolute error 3.61 p.p., R^2^ = 0.9892

Again, we performed identical analyses for the EpiMelt assays targeting the *APC*, *BRCA1*, and *H19* genes and the results were concordant (See S3–S7 Files).

### Dynamic range for the methylation quantification in MS-HRM

Most clinical samples, especially in oncology, contain a relatively small fraction of the pathologically altered tissue. After resection the sample may be contaminated with healthy tissues such as stroma or blood. Similarly, in liquid biopsies or in formalin fixed paraffin embedded (FFPE) material, a very small amount of the target template with altered methylation status is present due to the nature of the sample or extensive degradation of the material, respectively. Therefore, unless microdissection or enrichment of the template from malignant cells is performed, the methylation detection assays need to be calibrated for high sensitivity to allow detection of the template with altered methylation status in the vast background of the template from the healthy cells, or being present at a very low level in the test sample. Moreover, when using MIP primers in technologies such as MS-HRM the PCR bias phenomenon additionally hampers the sensitivity of the methylation detection.

**Fig 7:**
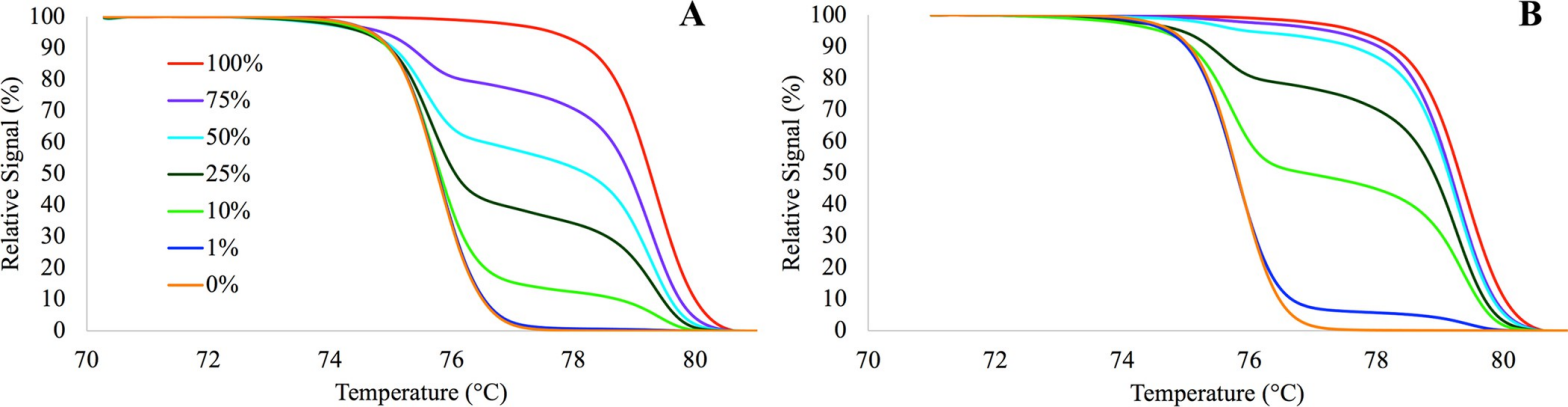
Example of HRM curves for the MS-HRM designed according to Wojdacz et al., and performed at low and high PCR annealing temperature. (A) low temperature, HRM curves representing 75%, 50%, 25% and 10% methylation levels are clearly distinguishable. (B) at high temperature only HRM curves representing 25%, 10% but at the same time the HRM curve representing 1% methylation level is now visible. The sensitivity of the assay is increased with the increased PCR annealing temperature but the dynamic range of the assay decreases.

As already mentioned in the introduction, we have previously developed a specific primer design strategy that allowed us to reverse PCR bias and increase the analytical sensitivity of the technologies that utilize MIP primers including MS-HRM [4]. When designing primers with this strategy, the sensitivity of e.g. the MS-HRM assay increases with the increase of the PCR annealing temperature [7]. However, at the same time the dynamic range of the assay narrows (Fig 7). Therefore, to be able to calculate the methylation level of the assay that was calibrated for high sensitivity (Fig 7, panel B), we needed to develop a procedure that allowed us to assess the dynamic range of the assay at the specific PCR conditions.

**Fig 8:**
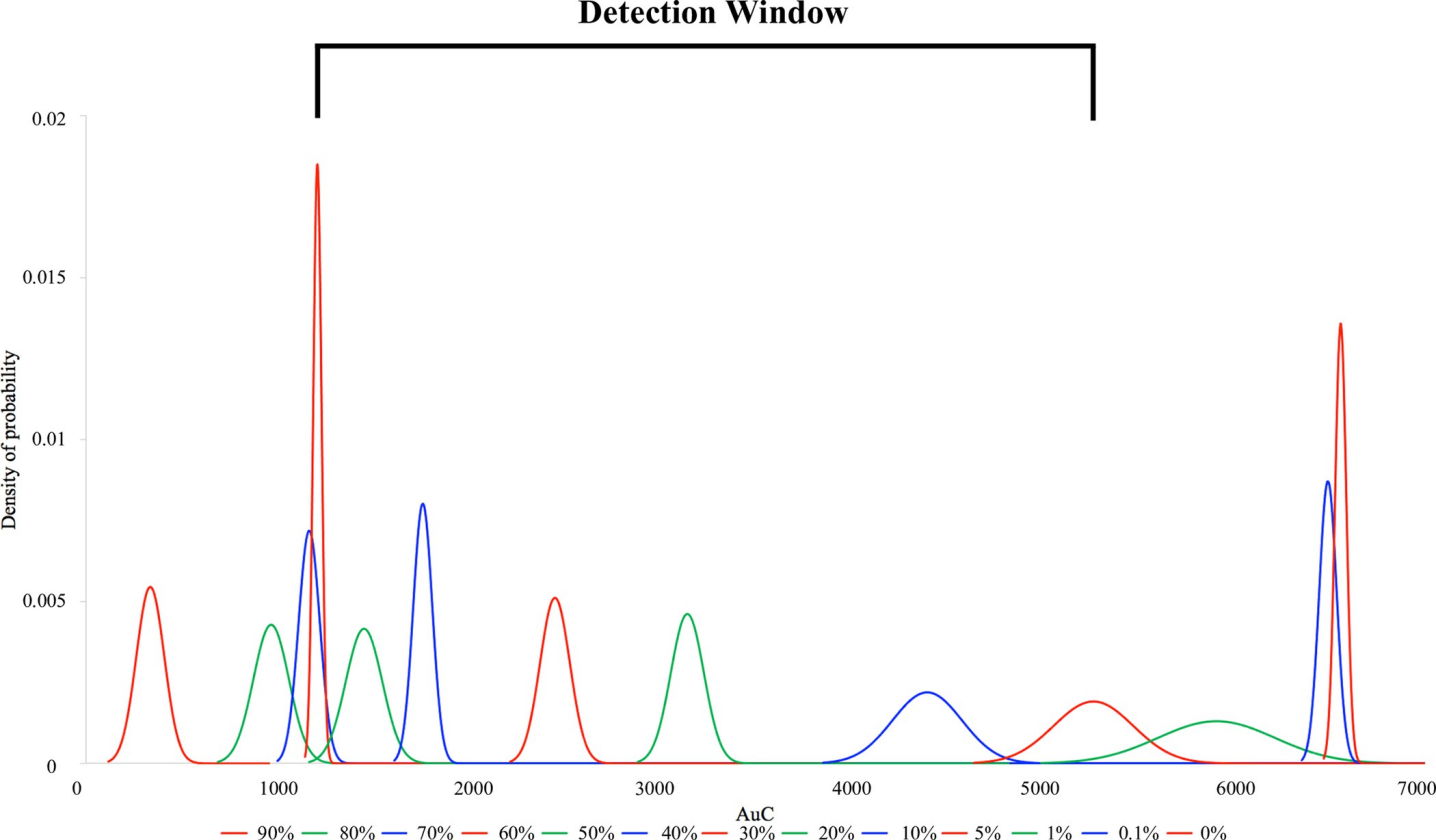
Example of calculation of the detection window for the EpiMelt *MGMT* assay. The bell-shaped Gaussian distribution for each control mix was calculated and only in the range where the bel-shaped density plots did not overlap within 2SD (marked in black) was considered the assay specific detection window and used for estimation of the methylation levels.

To find the dynamic range for a specific MS-HRM assay in each experiment, we calculated the mean and variance for each difference plot curve generated from the control mixes with known proportions of methylation. That allowed us to draw Gaussian distribution curves for each of the control mixes (Fig 8). We then only considered, for the methylation level quantification, the range of the standards where the Gaussian distribution bell-shaped curve (density of probability) did not overlap with 2SD (Fig 8 –marked in black) and named the range: Assay specific detection window.

An example of this type of data analysis is shown in Fig 8 for the EpiMelt *MGMT* assay. The detection window calculated for this assay at the annealing temperature of 59°C was in the range of 5–60% (Fig 8). In the detection window, we used the LSA procedure and Eq 3 to estimate the methylation levels of the unknown samples. The difference between estimated and expected methylation levels in unknown samples is displayed in Table 2 (right column). Again here, we observed up to 7 p.p. differences between expected and calculated methylation levels. However, the function assessing fitness of the expected and calculated methylation level in the sample had R2 = 0.9917, with MAE = 3.57 p.p, overall indicating an acceptable error of the calculation. (S3 File EpiMelt *MGMT* assay 59°C).

**Table 2:** Calculation of the methylation levels in the detection window for the EpiMelt MGMT assay.

Sample name	Expected methylation level	Calculated methylation level	Variation between expected and calculated methylation levels (Residuals)
Sample 50%	50%	48%	2 p.p
Sample 35%	35%	35%	0 p.p
Sample 15%	15%	19%	4 p.p
Sample 10%	10%	17%	7 p.p

Mean absolute error 3.57%, R^2^ = 0.9928

## Conclusion

In summary, we have established a procedure that with certain limitations allows for the calculation of methylation levels using Area Under the Curve (AUC), which is a derivate of the HRM curves. The major limitation of the method is accuracy. Most likely due to the PCR bias and technical variability observed in HRM based methylation screening experiments, our results showed that the accuracy of the method is about 10%. However, this is an acceptable limit in most of the methylation screening experiments. Our results also underlay the need to run a range of control dilutions in each MS-HRM experiment, and subsequently use those controls for the methylation levels assessment (and do not import controls from other runs). However, this approach is also recommended for e.g., gene expression quantification experiments.

If the primers for an MS-HRM assay were designed for high sensitivity, and according to the guidelines, we previously proposed, the dynamic range of the assay will be annealing temperature depended. In this case, we have proposed an automated calculation to assess the dynamic range in which the methylation levels of unknown samples can be estimated. The procedure can also be used to establish a cut-off for specific clinically or biologically relevant methylation level. The step-by-step description of data processing in the proposed procedure and excel templates enabling automatic calculations can be found in the supplementary section of the paper.

## Supporting information

S1 File Methylation levels calculator protocol. (PDF)

S2 File Methylation Levels Calculator (MLC). (XLSX)

S3 File EpiMelt *MGMT* assay analysis (59°C). (XLSX)

S4 File EpiMelt *APC* assay analysis (62°C). (XLSX)

S5 File EpiMelt *APC* assay analysis (65°C). (XLSX)

S6 File EpiMelt *H19* assay analysis (57°C). (XLSX)

S7 File EpiMelt *BRCA1* assay analysis (55°C). (XLSX)

S8 File EpiMelt *MGMT* assay raw text file_comma (59°C). (TXT)

S9 File EpiMelt *MGMT* assay raw text file_dot (59°C). (TXT)

S10 File EpiMelt *MGMT* assay raw text file_without outliers_comma (59°C). (TXT)

S11 File EpiMelt *MGMT* assay raw text file_without outliers_dot (59°C). (TXT)
