# Supplementary material for: Methylation levels assessment with Methylation-Sensitive High-Resolution Melting (MS-HRM)
Source: PLoS One. 2022 Sep 6;17(9):e0273058. doi: 10.1371/journal.pone.0273058 (PMC9447921; doi:10.1371/journal.pone.0273058)
Supplement: S1 File — (PDF) [file pone.0273058.s001.pdf]

Aug 16, 2022

Automated procedure for estimation of methylation levels in MS-HRM analysis

Sally Samsø Mathiasen[1], Jan Bińkowski[2], Tina Kjeldsen[1], Tomasz K Wojdacz [1, 2] , Lise Lotte Hansen [1]

[1]Department of Biomedicine, Aarhus University, Aarhus DK-8000, Denmark.;

[2]Independent Clinical Epigenetics Laboratory, Pomeranian Medical University, Szczecin, Poland;

1 Works for me Share

dx.doi.org/10.17504/protocols.io.n2bvj6yjxlk5/v1

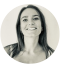

Sally Samsø Mathiasen

ABSTRACT

Testing for disease-related DNA methylation changes provides clinically relevant information in personalized patient care. Methylation-Sensitive High-Resolution Melting (MS-HRM) is a method used for measuring methylation changes and has already been used in diagnostic settings. This method utilizes one set of primers that initiate the amplification of both methylated and non-methylated templates. Therefore, the quantification of the methylation levels using MS-HRM is hampered by the PCR bias phenomenon. Some approaches have been proposed to calculate the methylation level of samples using the high-resolution melting (HRM) curves. However, limitations of the methylation calculation using MS-HRM have not been evaluated systematically and comprehensively. We used the Area Under the Curve (AUC), a derivative of the HRM curves, and least square approximation (LSA) to establish a procedure that allowed us to infer methylation levels in an MS-HRM experiment and assess the limitations of that procedure for the assays' specific methylation level measurement. The developed procedure allowed, with certain limitations, estimation of the methylation levels using HRM curves.

DOI

dx.doi.org/10.17504/protocols.io.n2bvj6yjxlk5/v1

PROTOCOL CITATION

Sally Samsø Mathiasen, Jan Bińkowski, Tina Kjeldsen, Tomasz K Wojdacz, Lise Lotte Hansen 2022. Automated procedure for estimation of methylation levels in MS-HRM analysis. **protocols.io**
https://protocols.io/view/automated-procedure-for-estimation-of-methylation-b3ptqmnn

Citation: Sally SamsÃÂ¸ Mathiasen, Jan BiÃÂkowski, Tina Kjeldsen, Tomasz K Wojdacz, Lise Lotte Hansen Automated procedure for estimation of methylation levels in MS-HRM analysis https://dx.doi.org/10.17504/protocols.io.n2bvj6yjxlk5/v1

This is an open access protocol distributed under the terms of the **Creative Commons Attribution License** (https://creativecommons.org/licenses/by/4.0/), which permits unrestricted use, distribution, and reproduction in any medium,

LICENSE

This is an open access protocol distributed under the terms of the Creative Commons Attribution License, which permits unrestricted use, distribution, and reproduction in any medium, provided the original author and source are credited

CREATED

Jan 11, 2022

LAST MODIFIED

Aug 16, 2022

PROTOCOL INTEGER ID

56787

DATA IMPORT

1 **MS-HRM data preparation for the analyses (when using Light Cycler system – other PCR systems may require adjusting of the data format)**

1.1 Normalize the HRM curves using the Gene Scanning software (we recommend default settings for the normalization).

1.2 Generate difference plots for each normalized melting curve with the 100% methylation melting curve as the baseline/reference.

1.3 If data for any of the samples contain obvious outliers, you may consider removing the outliers (at this point only note the name of the outlier).

1.4 Export the difference plot as a text file.
An example of layout of the file obtained from LightCycler480 instrument (Roche) see supplementary material S8-S11 File.

IMPORTANT: The example of the calculations performed with Methylation Level Calculator (MLC) in this protocol (S2 File Methylation Levels Calculator (MLC)) requires experimental layout as described in Plate set up section of the MLC template, for other sample layouts in the experiment remember to modify the columns and rows names accordingly.

Citation: Sally SamsÃÂ, Mathiasen, Jan BiÃÂkowski, Tina Kjeldsen, Tomasz K Wojdacz, Lise Lotte Hansen Automated procedure for estimation of methylation levels in MS-HRM analysis https://dx.doi.org/10.17504/protocols.io.n2bvj6yjxlk5/v1

This is an open access protocol distributed under the terms of the **Creative Commons Attribution License** (https://creativecommons.org/licenses/by/4.0/), which permits unrestricted use, distribution, and reproduction in any medium,

2 Methylation levels estimation procedure

2.1 Open the Methylation Levels Calculator (MLC) (S2 File Methylation Levels Calculator (MLC)).

> **IMPORTANT:** Make sure Excel solver Add-in software is installed and loaded.
> Mac: Go to Data > Analysis Tool > check Solver Add-in > OK.
> PC: Go to File > Options > Add-ins > Go button > check Solver Add-in > OK.

2.2 Open the exported text file exported from LC480 instrument (see for an example file: S10-S11 MGMT assay text_without outliers).

2.3 Select and copy all the data in the text file and paste it into the sheet “imported data” starting in cell B3. Make sure that the names in rows 2 and 3 agree (For the purpose of this example, the outliers are removed in the text file)

> **IMPORTANT:** To copy-paste the data form LC480 to excel it is important that the digital separator in the file exported from LC480 instrument and excel are the same.

> **IMPORTANT:** This procedure will only work for the sample plate layout presented in “plate set up” sheet of “S2 File Methylation Levels Calculator (MLC)”, for other sample layouts in the experiment that specific columns of the MLC need to be modified accordingly.

2.4 The MLC will now automatically calculate AUC and display the AUC value for

Citation: Sally SamsÃÂ¸ Mathiasen, Jan BiÃÂkowski, Tina Kjeldsen, Tomasz K Wojdacz, Lise Lotte Hansen Automated procedure for estimation of methylation levels in MS-HRM analysis https://dx.doi.org/10.17504/protocols.io.n2bvj6yjxlk5/v1

This is an open access protocol distributed under the terms of the **Creative Commons Attribution License** (https://creativecommons.org/licenses/by/4.0/), which permits unrestricted use, distribution, and reproduction in any medium,

each control and sample. That value is shown in raw 1 of the “Imported data” sheet of the MLC.

2.5 Check if the value of AUC for each replicate (replicates of each control are marked with the same colours) in row 1 agree within acceptable range (if not consider excluding outliers by replacing the values with 0. Compare the outlier with the findings of 1.4).

IMPORTANT: If the MLC do not automatically perform the subsequent calculation, then change the calculation options to automatic in Excel. (Go to Formulas > Calculation options > Automatic)

2.6 Go to the sheet: “0 variable”.

Here MLC automatically performs subsequent calculations:
- Panel 1: The AUC for each of the replicates of controls is calculated from the data imported.
- Panel 2: The equation 1 is used to calculate theoretical AUC for each methylation level starting from the maximum AUC in this experiment which is AUC for the 0% methylation level (AUC of 100% control was chosen as reference for the calculation of difference plots thus 0% control has maximum AUC).
- Panel 3: Theoretical standard curve (blue) and obtained values of AUC (red) for controls in the experiment are plotted.

2.7 Go to the sheet: “1 variable”.

Here MLC automatically performs subsequent calculations:
- Panel 1: The AUC for each of the replicates of controls is calculated from the data imported.
- Panel 2: The equation 2 is used to calculate theoretical AUC for each methylation level with M value set to 1.
- Panel 3: Theoretical standard curve (blue) and obtained values of AUC (red) for controls in the experiment are plotted.

Citation: Sally SamsÃÂ¸ Mathiasen, Jan BiÃÂkowski, Tina Kjeldsen, Tomasz K Wojdacz, Lise Lotte Hansen Automated procedure for estimation of methylation levels in MS-HRM analysis https://dx.doi.org/10.17504/protocols.io.n2bvj6yjxlk5/v1

This is an open access protocol distributed under the terms of the **Creative Commons Attribution License** (https://creativecommons.org/licenses/by/4.0/), which permits unrestricted use, distribution, and reproduction in any medium,

2.8 Go to the sheet: “1 variable after LSA”.

Add-in Solver needs to be used here.

2.9 Go to: Data > Solver > Solve on the pop-up window > OK in the next pop-up window. Now the M value has been calculated by LSA and the standard curve was recalculated accordingly - the blue curve in fig1 in panel 3 sheet “1 variable”.

Here MLC automatically performs subsequent calculations:
- Panel 1: The AUC for each of the replicates of controls is calculated from the data imported.
- Panel 2:The equation 2 is used to calculate theoretical AUC for each methylation level with M value recalculated by LSA.
- Panel 3: The standard curve is recalculated (blue) and obtained values of AUC (red) for controls in the experiment are plotted.

2.10 Go to the sheet: “2 variables”

Here MLC automatically performs subsequent calculations:
- Panel 1: The AUC for each of the replicates of controls is calculated from the data imported.
- Panel 2:The equation 3 is used to calculate theoretical AUC for each methylation level with N value set to 1
- Panel 3: The standard curve is recalculated (blue) and obtained values of AUC (red) for controls in the experiment are plotted.

2.11 Go to the sheet: “2 variables after LSA”.

Add-in Solver needs to be used here.

2.12 Go to: Data > Solver > Solve on the pop-up window and click OK in the next pop-

Citation: Sally SamsÃÂ, Mathiasen, Jan BiÃÂkowski, Tina Kjeldsen, Tomasz K Wojdacz, Lise Lotte Hansen Automated procedure for estimation of methylation levels in MS-HRM analysis https://dx.doi.org/10.17504/protocols.io.n2bvj6yjxlk5/v1

This is an open access protocol distributed under the terms of the **Creative Commons Attribution License** (https://creativecommons.org/licenses/by/4.0/), which permits unrestricted use, distribution, and reproduction in any medium,

up window. Now the M and N value have been calculated by LSA and standard curve was recalculated accordingly - the blue curve in fig 1 in panel 3 sheet “2 variable”.

Here MLC automatically performs subsequent calculations:
- **P**anel 1**:** The AUC for each of the replicates of controls is calculated from the data imported.
- Panel 2:The equation 3 is used to calculate theoretical AUC for each methylation level with M and N values recalculated by LSA.
- Panel 3: Theoretical standard curve (blue) and obtained values of AUC (red) for controls in the experiment are plotted.

ESTIMATION OF THE METHYLATION LEVEL IN UNKNOWN SAMLES

3 ESTIMATION OF THE METHYLATION LEVEL IN UNKNOWN SAMLES

The MLC uses polynomial trend function to automatically read the methylation levels from the standard curve obtained in section 2

3.1 Go to the sheet: “Polynomial trend function (PTF)”

Here MLC automatically performs subsequent calculations:
- Panel 1: The theoretical standard curve previously calculated describes AUC as a function of methylation level (equation 3). To use excel for calculation (technical limitation of the program) of the methylation level of unknown samples the theoretical standard curve calculated in “2 variables after LSA” needs to be transformed to describe methylation level as a function of AUC.
- Panel 2: Polynomial trend function with 10 degrees describes the methylation level as a function of AUC. The polynomial trend function describing the standard curve obtained with equation 3 is calculated and plotted (red).

3.2 Go to the sheet: “Unknown sample calculation (USC)”

Citation: Sally SamsÃÂ¸ Mathiasen, Jan BiÃÂkowski, Tina Kjeldsen, Tomasz K Wojdacz, Lise Lotte Hansen Automated procedure for estimation of methylation levels in MS-HRM analysis https://dx.doi.org/10.17504/protocols.io.n2bvj6yjxlk5/v1

This is an open access protocol distributed under the terms of the **Creative Commons Attribution License** (https://creativecommons.org/licenses/by/4.0/), which permits unrestricted use, distribution, and reproduction in any medium,

Here MLC automatically performs subsequent calculations:
- Panel 1.1: The sample name
- Panel 1.2: The AUC for each of the replicates of the samples are calculated from the data imported.
- Panel 1.3: The methylation level of the unknown samples calculated by equation 3 with M and N variables (using transformation described in sheet: "Polynomial trend function (PTF)".

CALCULATION OF THE EXPRIMENT SPECIFIC DETECTION WINDOW

4 CALCULATION OF THE EXPRIMENT SPECIFIC DETECTION WINDOW

4.1 Go to the sheet: "Cut off (CO)"

Here MLC automatically performs subsequent calculations (this operation may take a bit of time):
- Panel 1.1 and panel 1.2: The AUC for each of the replicates of controls is calculated from the data imported.
- Panel 1.3: The Standard deviation for each of the replicates of controls is calculated
- Panel 1.4: The mean value for each of the replicates of controls is calculated
- Panel 2: The normal distribution/Gaussian distribution for each of the controls is plotted.

4.2 Go to sheet: "Detection window"

Here MLC automatically performs subsequent calculations:
- Panel 1.1 and panel 1.2: The AUC for each of the replicates of controls is calculated from the data imported.
- Panel 1.3: The Standard deviation for each of the replicates of controls is calculated
- Panel 1.4: The mean value for each of the replicates of controls is calculated
- Panel 2: The overlap between consecutive controls is calculated.
- Panel 2.1: List of the controls in consecutive order.
- Panel 2.2: The overlap between consecutive controls is highlighted in

Citation: Sally SamsÃÂ, Mathiasen, Jan BiÃÂkowski, Tina Kjeldsen, Tomasz K Wojdacz, Lise Lotte Hansen Automated procedure for estimation of methylation levels in MS-HRM analysis https://dx.doi.org/10.17504/protocols.io.n2bvj6yjxlk5/v1

This is an open access protocol distributed under the terms of the **Creative Commons Attribution License** (https://creativecommons.org/licenses/by/4.0/), which permits unrestricted use, distribution, and reproduction in any medium,

pink.
- Panel 3: **IMPORTANT:** Fill in lower and higher limit (use %) of the detection window in cell P7 and Q7 based on the calculations from Panel 2, (in this example: upper limit: 60% and 50% and lower limit: 10% and 5%).

5 CALCULATION OF METHYLATION LEVELS IN THE ASSAY SPECIFIC DETECTION WINDOW

5.1 Go to the sheet: “2 variables within DW”.

IMPORTANT: If the MLC do not automatically perform the subsequent calculation, then change the calculation options to automatic in Excel. (Go to Formulas > Calculation options > Automatic)

Add-in Solver needs to be used here.

5.2 Go to: Data > Solver > Solve on the pop-up window and click OK in the next pop-up window. Now the M and N value have been calculated by LSA and standard curve was recalculated accordingly.

Here MLC automatically performs subsequent calculations:
- Panel 1**:** The AUC for each of the replicates of controls is calculated from the data imported.
- Panel 2:The equation 3 is used to calculate theoretical AUC for each methylation level with M and N values recalculated by LSA.
- Panel 3: Theoretical standard curve (blue) and obtained values of AUC (red) for controls in the experiment are plotted.

5.3 Go to the sheet “PTF within DW”. PTF (Polynomial Trend Function).

Citation: Sally SamsÃÂ, Mathiasen, Jan BiÃÂkowski, Tina Kjeldsen, Tomasz K Wojdacz, Lise Lotte Hansen Automated procedure for estimation of methylation levels in MS-HRM analysis https://dx.doi.org/10.17504/protocols.io.n2bvj6yjxlk5/v1

This is an open access protocol distributed under the terms of the **Creative Commons Attribution License** (https://creativecommons.org/licenses/by/4.0/), which permits unrestricted use, distribution, and reproduction in any medium,

Identical as **3.1** but within the detection window.

Here MLC automatically performs subsequent calculations:
- Panel 1: The theoretical AUC calculated from equation 3 using M and N values calculated by Excel Add-in Solver for the whole range and in the detection window.
- Panel 2: The theoretical standard curve (blue) and a polynomial trendline with 10 degrees.

5.4 Go to the sheet "USC within DW" USC (Unknown Sample Calculations)

Here MLC automatically performs subsequent calculations:
- Panel 1.1: The sample name
- Panel 1.2: The AUC for each of the replicates of the samples is calculated from the data imported.
- Panel 1.3: The methylation level of the unknown samples calculated by equation 3 with M and N variables in the assay specific detection window.

Citation: Sally SamsÃÂ¸ Mathiasen, Jan BiÃÂkowski, Tina Kjeldsen, Tomasz K Wojdacz, Lise Lotte Hansen Automated procedure for estimation of methylation levels in MS-HRM analysis https://dx.doi.org/10.17504/protocols.io.n2bvj6yjxlk5/v1

This is an open access protocol distributed under the terms of the **Creative Commons Attribution License** (https://creativecommons.org/licenses/by/4.0/), which permits unrestricted use, distribution, and reproduction in any medium,
